# *Calosoma aethiops* (Jeannel, 1940) as a new synonym of *Calosoma imbricatum hottentotum* Chaudoir, 1852, a new status of *Calosoma roeschkei* Breuning, 1927, and a revision of the *Calosoma senegalense* group sensu Häckel, 2012 (Coleoptera, Carabidae, Carabini)

**DOI:** 10.3897/zookeys.609.6822

**Published:** 2016-08-08

**Authors:** Martin Häckel, Jan Farkač, Rostislav Sehnal

**Affiliations:** 1 Department of Game Management and Wildlife Biology, Faculty of Forestry and Wood Sciences, Czech University of Life Sciences, Kamýcká 1176, CZ-165 21 Prague 6 – Suchdol, Czech Republic; 2 V Zahrádkách 962, CZ-273 51 Unhošť, Czech Republic

**Keywords:** Carabidae, Carabinae, Calosoma, new synonymy, Africa

## Abstract

*Calosoma aethiops* (Jeannel, 1940) as a new synonym of *Calosoma imbricatum hottentotum* Chaudoir, 1852, a new status of *Calosoma roeschkei* Breuning, 1927, and a revision of the *Calosoma senegalense* group sensu Häckel, 2012 (Coleoptera: Carabidae: Carabini). Conducted is a taxonomic revision of the *Calosoma senegalense* group sensu Häckel, 2012. Placed in the group sensu stricto are four species: *Calosoma planicolle* Chaudoir, 1869, *Calosoma scabrosum* Chaudoir, 1843, *Calosoma senegalense* Dejean, 1831, and *Ctenosta strandi* Breuning, 1934. *Calosoma aethiops* Jeannel, 1940 is synonymized with *Calosoma imbricatum hottentotum* Chaudoir, 1852, and *Calosoma roeschkei* Breuning, 1927 is newly regarded as a subspecies of *Calosoma scabrosum*. The taxonomic conclusions are based on morphometry of the holotypes and 10 male and 10 female specimens of each taxon, and on morphology of the aedeagus including inflated endophalus.

## Introduction

*Calosoma* is the second most speciose genus of the subfamily Carabinae, with 168 (Lorenz 2005), 128 (Bruschi 2013) or 129 (Häckel 2013) species described from all zoogeographic regions. Most species are excellent fliers widely distributed on all continents and numerous islands, but some are secondarily brachypterous or apterous with narrow distributions (Bruschi 2013, Häckel 2012). Some species inhabit more zoogeographic regions, and some extend to a neighboring continent that belongs in the same zoogeographic region (Häckel 2012). Examples of such distributions are some species of the *Calosoma maderae* group (*Calosoma imbricatum* subgroup sensu Häckel 2013) and of the *Calosoma senegalense* group (*Calosoma senegalense* subgroup sensu Häckel 2013), which inhabit the area of the Horn of Africa. The distributions are probably to some extent responsible for the unsettled situation in the species-level taxonomy of the group (Bruschi 2013, Häckel 2012, 2013, Häckel & Farkač 2012), a part of which this paper attempts to resolve. At the same time it respects the recent supraspecific classification (Häckel 2013), which in light of the known genetic analyses (Su et al. 2005) does not support the traditional subgeneric divisions.

## Material and methods

The classification of the group is based primarily on external structural details of the adult, with species-level taxonomy relying also on structural details of the expanded endophalus. The aedeagi were dissected, preserved, studied dry and glued on cards appended beneath the dissected specimens. For study of the endophalus, the aedeagus was soaked 48 hours in 1:1 solution of water and 8% acetic acid, and then the endophalus was inflated using a small Heavy Duty (12V) compressor normally used to inflate tires, set at medium pressure. Fixation of the endophalus morphology was secured by slow drying on a portable electric (220V) single-plate heater, and the whole aedeagus-endophalus preparation was then glued on a paper card. The preparations were photographed by Canon G10 Digital Compact in macrophoto regime with flash. Aedeagi were photographed in the right lateral view, with details of their tips also slanted at an angle.

Inspected and evaluated were the following morphometric parameters of the holotypes and 20 samples (10 males and 10 females) of each species:

a) total length of the adult including mandibles (TL),

b) maximum head width including eyes to maximum pronotal width ratio (WP/WH),

c) maximum pronotal length to maximum width ratio (WP/LP),

d) maximum elytral length to maximum width ratio (LE/WE).

Subjective evaluations included also differences in termination of the aedeagus (apex) and of the inflated endophalus. Measured were morphometric parameters of 10 males and 10 females of the following taxa: *Calosoma planicolle*, *Calosoma scabrosum scabrosum*, *Calosoma scabrosum roeschkei*, *Calosoma senegalense*, *Ctenosta strandi* (all taxa belong to *Calosoma senegalense* group), and *Calosoma imbricatum imbricatum* from populations inhabiting the Afrotropical region (including Oman and Yemen on the Arab peninsula) and *Calosoma imbricatum hottentotum* (*Calosoma maderae* group, *Calosoma imbricatum* subgroup). Measured holotypes include *Calosoma scabrosum* (Chaudoir), *Calosoma scabrosum roeschkei* (Breuning), *Calosoma imbricatum hottentotum* (Chaudoir) and *Calosoma aethiops* (Jeannel).

The material examined is housed in the collections listed below:

cMNHN
 Muséum national d’Histoire naturelle, Paris, France 


cNBCL
 National Biodiversity Center, Leiden, Netherlands 


cNMP
 Národní muzeum, Prague, Czech Republic 


cFAR
 Jan Farkač collection, Prague, Czech Republic 


cHAC
 Martin Häckel collection, Hostivice, Czech Republic 


cSEH
 Rostislav Sehnal collection, Unhošť, Czech Republic 


cWRA
 David W. Wrase collection, Berlin, Germany 


## Systematic part

### *Calosoma senegalense* species group (sensu Häckel 2013):

*Calosoma senegalense* subgroup (= *Calosoma senegalense* group s. str.)

#### Calosoma (Calosoma) planicolle

Taxon classificationAnimaliaColeopteraCarabidae

Chaudoir, 1869

Plate 4: Fig. 4

Calosoma planicolle Chaudoir, 1869: 369 (type loc. “près du Zambéze”, type in cMNHN).
Calosoma procerum Harold, 1880: 260 (type loc. “Taita District, Kenya Colony, Ukamba”), syn. sn. Breuning 1927: 188.
Calosoma (Ctenosta) planicolle Breuning, 1927: 188. Lapouge 1932: 415; Deuve 1978: 250; Culot 1990: 9; Lorenz 2005: 68; Bruschi 2013: 132.
Ctenosta (s. str.) planicolle Jeannel, 1940: 130. Rougemont 1976: 248; Vigna Taglianti and Bruschi 1986: 22.
Calosoma (s. str.) planicolle Häckel, 2013: 30.

##### Material studied.

**BOTSWANA.** 1♂, 3♀: Ngamiland district, ne. of Maun, Tamalakane (cHAC).

**KENYA.** 1♂, 1♀: Tsavo, Mtitoanday (cSEH); 1♂, 1♀: Eastern 729, Sosoma, 202 km E of Thika (cSEH).

**MOZAMBIQUE.** 1♀: Sofala province, 30 km S Caia (cSEH).

**Namibia.** 1♂: Kavango reg., Okavango river, Rundu, 1050 m (cHAC); 1♂, 1♀: Caprivi reg., Bagani- Popa Falls (cHAC).

**Zambia.** 1♂: Southern Prov., Livingstone env., Victoria Falls (cHAC).

**Zimbabwe.** 1♂, 2♀: Midlands Prov., Kwekwe env. 20 km w. Ngezi Park (cHAC); 1♂, 1♀: Matabeleland Prov., 60 km N of Bulawayo, Marapoosa road (cHAC); 2♂: Masvingo province, 95 km NE Beitbridge, Bubi river (cSEH)

##### Distribution.

Angola, Botswana, Democratic Congo, Ethiopia, Kenya, Lesotho, Madagascar, Malawi, Mozambique, Republic of South Africa, Somalia, Tanzania, Swaziland, Uganda, Zambia, Zimbabwe.

#### Calosoma (Calosoma) scabrosumscabrosum

Taxon classificationAnimaliaColeopteraCarabidae

Chaudoir, 1869

**Plate 1.**
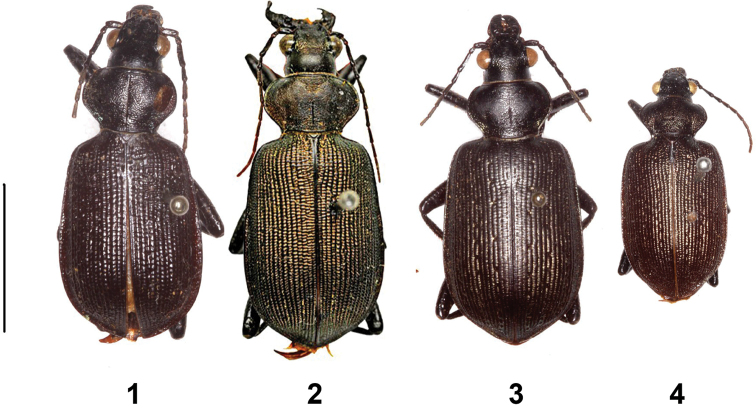
Type material (habitus in dorsal aspect, male). Scale bar equals 10 mm. **1** *Calosoma scabrosum scabrosum* (holotype) **2** *Calosoma scabrosum roeschkei* (holotype) **3** *Calosoma imbricatum hottentottum* (holotype of *Ctenosta aethiops* Jeannel, 1940) **4** *Calosoma imbricatum hottentottum* (holotype).

**Plate 2.**
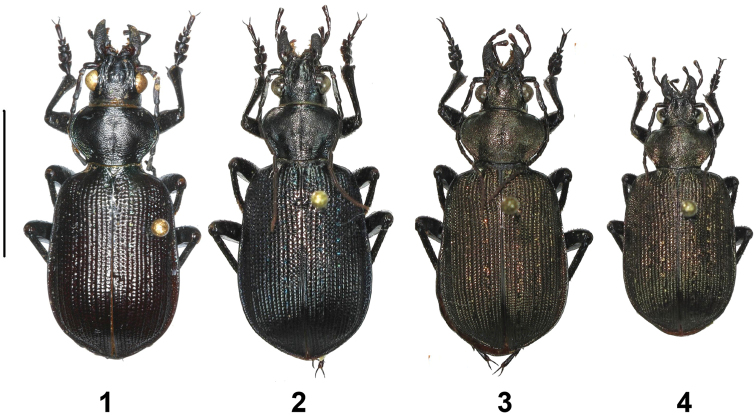
Material studied (habitus in dorsal aspect, male). **1** *Calosoma scabrosum scabrosum* (Djibouti: Obock) **2** *Calosoma scabrosum scabrosum* (Oman: Dhofar, holotype of *Calosoma jakli* Häckel, Farkač & Sehnal, 2005) **3** *Calosoma scabrosum roeschkei* (Kenya: Voi env.) **4** *Calosoma imbricatum hottentottum* (Namibia: Okahandja). Scale bar equals 10 mm.

Plate 1 : Fig. 1. Plate 2 : Figs 1–2. Plate 4. Fig. 1.

Calosoma scabrosum Chaudoir, 1843: 745 (type loc. “Kordofan”).
Calosoma kordofanum [Kollar in litt.] syn. sn. Chaudoir 1852: 100.
Calosoma (Ctenosta) scabrosum Breuning, 1927: 185. Lapouge 1932: 414; Deuve 1978: 250; Culot 1990: 9; Lorenz 2005: 68; Bruschi 2013: 127.
Ctenosta (s. str.) scabrosum Jeannel, 1940: 128. Rougemont 1976: 247; Vigna Taglianti and Bruschi 1986: 21.
Calosoma (Ctenosta) jakli Häckel, Farkač & Sehnal, 2005: 2 (type loc. “Oman: Dhofar”), syn. sn. Häckel et al., 2010: 11.
Calosoma (s. str.) scabrosum Häckel, 2012: 57. Häckel 2013: 31.

##### Type material.

***Calosoma scabrosum* Chaudoir, 1869.** 1♂ labelled “HOLOTYPE / Ex Musaeo Chaudoir / Ctenosta scabrosum (Chd.) P. Basilewsky vid. 1992 (cMNHN); 1♂ labelled “SW Asia, S Oman, Dzhopar Prov., Al Mughsayi vill. env., 0–50 m a.s.l., VIII.1999, lgt. S. Jákl / HOLOTYPE Calosoma jakli det. Häckel, Farkač & Sehnal, 2005 / Calsoma scabrosum det. Häckel, Farkač & Sehnal, 2010” (cHAC).

##### Other material studied.

**Djibouti.** 1♂: “Obock” (cNMP).

**Oman.** 5♀: Dzhophar Prov., Takwa env., 50 m a.s.l. (cFAR, cHAC, cSEH); 1♀: rd. Al Mughsayi – Salalah, ca 3 km from Mughsayi, 20 m a.s.l. (cFAR); 1♀: Dhophar Province, Takwa env., 200 m a.s.l. (cKAL); 3♂, 2♀: Dzhofar prov., Wadi Nashib, 24 km E Salalah (cHAC, cSEH); 5♂: Dzhofar prov., Wadi Nashib, 20 km E Salalah (cHAC, cSEH).

**SENEGAL** (Niger or Chad probably). 1♀: ”Senegal” (cNMP).

**Yemen.** 1♂: NW Al Mukhallā: N: 14°37‘;/ E: 49°03‘ Kawr Saybān Mtn. (cHÄC).

##### Distribution.

Chad, Djibouti, Eritrea, Ethiopia, Kenya, Niger, Nigeria, Oman, Somalia, South Sudan, Tanzania, Yemen. Data from Burundi, Rwanda and Uganda need confirmation.

#### Calosoma (Calosoma) scabrosumroeschkei

Taxon classificationAnimaliaColeopteraCarabidae

Breuning, 1927

**Plate 3.**
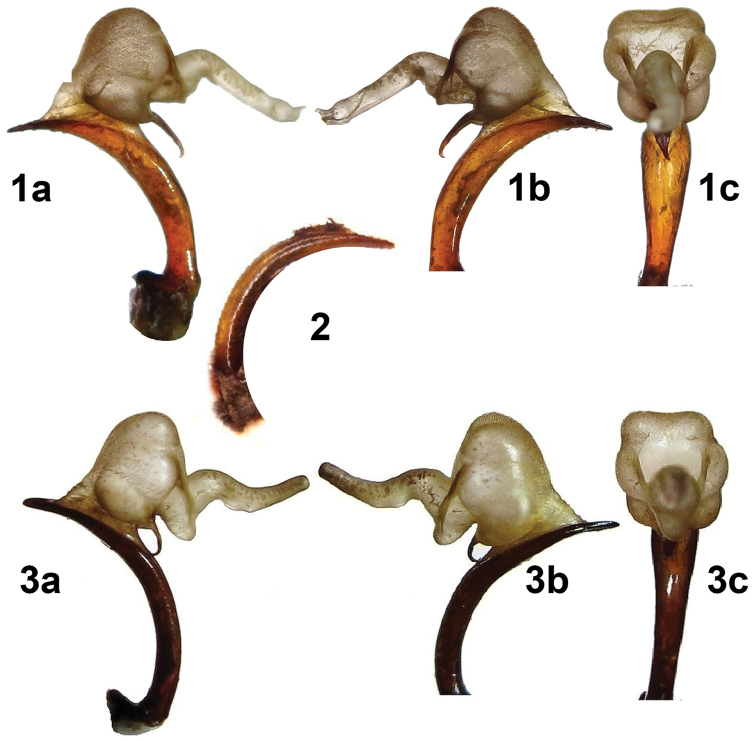
Aedeagi of *Calosoma imbricatum hottentotum* (Namibia) and *Calosoma scabrosum roeschkei* (Kenya) compared with aedeagus of “*Calosoma aethiops*” (holotype). **1** *Calosoma imbricatum hottentottum* (Namibia: Okahandja); a – aedeagus with expanded endophalus in right lateral view, b – the same in left lateral view, c – the same in anterior view **2** *Calosoma imbricatum hottentottum* (holotype of *Ctenosta aethiops* Jeannel, 1940) aedeagus in left lateral view **3** *Calosoma scabrosum roeschkei* (Kenya: Voi env.), a – aedeagus with expanded endophalus in right lateral view, b – the same in left lateral view, c – the same in anterior view.

**Plate 4.**
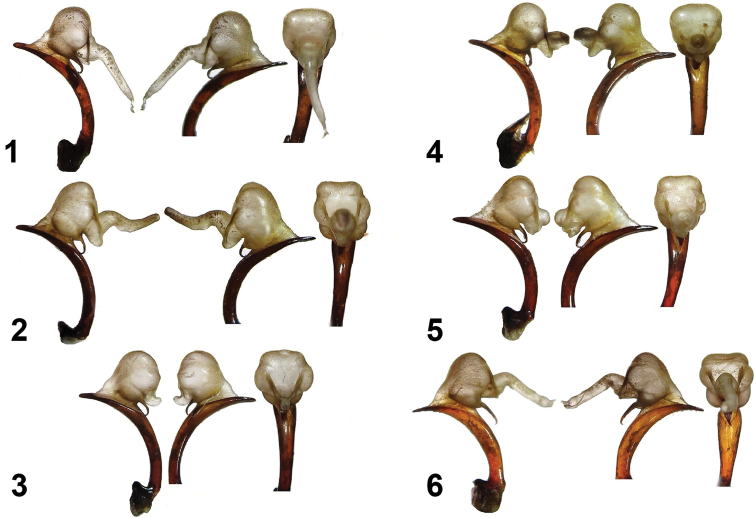
Aedeagi of *Calosoma senegalense* species group (sensu Häckel 2012: 30) and *Calosoma imbricatum hottentotum* (Namibia). a – aedeagus with expanded endophalus in right lateral view, b – same in left lateral view, c – same in anterior view **1** *Calosoma scabrosum scabrosum* (Oman: Dhofar) **2** *Calosoma scabrosum roeschkei* (southeastern Kenya: Voi env.) **3** *Ctenosta strandi* (northeastern Kenya: El Wak) **4** *Calosoma planicolle* (Namibia: Kavango) **5** *Calosoma senegalense* (Zimbabwe: Shamva) **6** *Calosoma imbricatum hottentottum* (Namibia: Okahandja).

Plate 1 : Fig. 2. Plate 2 : Fig. 3. Plate 3 : Fig. 3. Plate 4: Fig. 2.

Calosoma (Ctenosta) scabrosum roeschkei Breuning, 1927: 185 (type loc. “Usambara”).
Ctenosta (s. str.) aethiops (partim) Jeannel, 1940: 128 (loc. “Diré-Daoua”); Rougemont 1976: 247. Vigna Taglianti and Bruschi 1986: 21.
Ctenosta (s. str.) orientale (partim) Jeannel, 1940: 129 (loc. “Érythrée: Tessenei”).
Ctenosta (s. str.) scabrosum var. roeschkei Jeannel, 1940: 128.
Calosoma (Ctenosta) aethiops Culot, 1990: 9. Lorenz 2005: 68.
Calosoma (s. str.) scabrosum Häckel, 2012: 57. Häckel 2013: 31.
Calosoma (Ctenosta) roeschkei Bruschi, 2013: 129.

##### Type material.

*Calosoma roeschkei* Breuning, 1927. 1♂ labelled “Usambara”(cNBCL).

##### Other material studied.

**Kenya.** 1♂, 1♀: E of Garsen, W of Witu (cSEH); 2♂, 1♀: S of Voi (cHAC); 1♂, 1♀: Taita prov. Sagala Hills, Voi env. (cHAC); 1♂: Tsavo East, Voi Lodge, 3.23S/38.34E (cWRA); 1♀: NE prov. El Wak (cHAC); 1♂, 1♀: Modo Gashi to Wajir (cHAC); 2♂, 2♀: Coast province, Garissa, N of Bura (cHAC, cSEH), 1♂: Amboseli National Park (cSEH); 2♀: Eastern 729, Sosoma, 202 km E of Thika (cSEH).

**Sudan.** 1♂, 1♀: Vad Medani (cSEH).

##### Geographic distribution.

Chad, Kenya, Somalia, Sudan, Tanzania.

#### Calosoma (Calosoma) senegalense

Taxon classificationAnimaliaColeopteraCarabidae

Dejean, 1831

Plate 4: Fig. 5

Calosoma sengalense Dejean, 1831: 562 (type loc. “Sénegal”).
Calosoma mossambicense Klug, 1853: 247 (type loc. “Téte”). Calosoma (Ctenosta) senegalense mossambicense Breuning, 1927: 187.
Ctenosta senegalense Motschulsky, 1865: 306.
Calosoma (Ctenosta) senegalense Breuning, 1927: 187. Lapouge 1932: 415. Deuve 1978: 247; Culot 1990: 9; Lorenz 2005: 68; Bruschi 2013. 131.
Ctenosta (s. str.) senegalense Jeannel, 1940: 129. Rougemont 1976: 248; Vigna Taglianti and Bruschi 1986: 21.
Calosoma (s. str.) senegalense Häckel, 2012: 58, 64; 2013: 31.

##### Material studied.

**Botswana.** 1♂, 1♀: Ngamiland district, ne. of Maun, Tamalakane (cHAC).

**Ethiopia.** 1♂, 1♀: Gambela region, Gambela env., 400 m (cHAC).

**Ghana.** 1♂, 1♀: Northern Prov., West Gonja district, Damongo env. (cHAC).

**Kenya.** 1♂, 1♀: Coast Prov., Taita-Taveta Co., s. of Voi (cHAC).

**Madagascar.** 1♂, 2♀: Toliara prov., Ampanihy district, Ejeda env. (c FAR, cHAC); 1♂, 1♀: Mahajanga Prov., Ampatika env., Mahajamba river (cHAC).

**Namibia.** 1♂: Caprivi reg., Bagani- Popa Falls; 1♀: Khomas region, 40 km e. Windhoek (airport) (cHAC).

**Senegal.** 1♂, 1♀: Thiès region, M’bour department, Saly Portudal (cHAC).

**Tanzania.** 1♂: Arusha reg., Mto Wa Mbu env. (cHAC); 1♀: Morogoro region, Mikumi (cHAC). **Zimbabwe.** 1♂: 20 km NE Shamva, Nyagui river (cHAC).

##### Distribution.

Angola, Benin, Botswana, Burkina Faso, Burundi, Cameroon, Cabo Verde Islands, Chad, Congo, Côte ďIvoire, Democratic Congo, Eritrea, Ethiopia, Gabon, Gambia, Ghana, Guinea, Guinea-Bissau, Guinea Equatorial, Kenya, Lesotho, Liberia, Madagascar, Malawi, Mali, Mauritania, Mozambique, Namibia, Niger, Nigeria, Republic of Central Africa, Republic of South Africa, Rwanda, Senegal, Sierra Leone, Somalia, Swaziland, Tanzania, Togo, Uganda, Zambia, Zimbabwe.

#### Calosoma (Calosoma) strandi

Taxon classificationAnimaliaColeopteraCarabidae

Breuning, 1934

Plate 4: Fig. 3.

Calosoma (Ctenosta) strandi Breuning, 1934: 38 (type loc. “Masaua”). Culot 1990: 9; Lorenz 2005: 68; Bruschi 2013: 130.
Ctenosta (s. str.) strandi Jeannel, 1940: 130. Rougemont 1976: 244; Vigna Taglianti and Bruschi 1986: 21.
Calosoma (s. str.) strandi Häckel, 2013: 31.

##### Material studied.

**Kenya.** 8♂, 6♀: North-Eastern Prov., El Wak. (cFAR, cHAC, cSEH); 2♂, 4♀: Eastern Prov. Marsabit to South Horq (cFAR, cHAC, cSEH).

##### Distribution.

Eritrea, Ethiopia, Kenya, Somalia.

### *Calosoma maderae* species group (sensu Häckel 2013)

*Calosoma imbricatum* subgroup

#### Calosoma (Calosoma) imbricatumimbricatum

Taxon classificationAnimaliaColeopteraCarabidae

Klug, 1832

Calosoma imbricatum Klug, 1832: pl. IX. (type loc. “Cap Vert”). Calosoma (Caminara) imbricatum Breuning, 1927: 221. Caminara (Caminara) imbricata Lapouge 1932: 410, Jeannel 1940: 104. Culot 1990: 7; Lorenz 2005: 69; Bruschi 2013: 76, Häckel 2013: 24.
Caminara arabica Motschulsky, 1865: 304. Caminara imbricata arabica Lapouge 1932: 410.
Calosoma (Caminara) loffleri Mandl, 1953: 57.
Calosoma (Caminara) loffleri m. *rufoapendiculata* Mandl, 1967: 44.
Calosoma (Caminara) linnavouri Mandl, 1970: 61.

##### Material studied.

**Kenya.** 1♂, 1♀: Marsabith to South Orr (cHAC).

**Oman.** 8♂, 8♀: Wadi Qitbit, 150m (cFAR, cHAC, cSEH).

**Senegal.** 1♀: Senegal (cHAC).

**Sudan.** 1♂: Port Sudan (cHAC).

##### Distribution.

Algeria, Cabo Verde Islands, Canarian Islands, Chad, Djibuti, Egypt, Eritrea, Ethiopia, western India, Iran, Iraq, northern Kenya, Kuwait, Libya, Mali, Niger, Oman, Pakistan, Saudi Arabia, Senegal, Somalia, Sudan, United Arab Emirates, Yemen.

#### Calosoma (Calosoma) imbricatumhottentotum

Taxon classificationAnimaliaColeopteraCarabidae

Chaudoir, 1852

Plate 1 : Figs 3–4. Plate 2 : Fig. 4. Plate 3 : Figs 1–2. Plate 4. Fig. 6.

Calosoma hottentotum Chaudoir, 1852: 99 (type loc. “Cap de bonne-Espérance”). Deuve 1978: 249.
Calosoma (Caminara) imbricatum hottentotum Breuning, 1927: 221. Mandl 1970: 61, 62; Culot 1990: 7; Lorenz 2005: 69; Bruschi 2013: 76.
Caminara (Caminara) imbricata hottentota Lapouge, 1932: 410. Jeannel 1940: 105.
Ctenosta (s. str.) aethiops Jeannel, 1940: 127 (type loc. “Azbin, à 20 km. ďAgadès, dans ľAïr”), **new synonym.**
Calosoma (s. str.) imbricatum hottentotum Häckel, 2013: 24.

##### Type material.

1♂ labelled “LECTOTYPE / Ex Musaeo Chaudoir / Calosoma hottentotum Lectotype Chaud. 1852 Th. Deuve det,. 1978” (cMNHN); 1♂ labelled “MUSEUM PARIS AZBIN (AIR) REG. de TINTABORAC 20 K E ď AGADÈS CAPde POSTH 1908 / HOLOTYPE / aethiops n. sp. Jeannel det.” (cMNHN).

##### Other material studied.

**Kenya.** 1♂, 1♀: Amboseli National Park (cSEH).

**Namibia.** 2♂, 2♀: Omaruru (cHAC); 5♂, 3♀: Okahandja, Gross Bamen (cHAC, cSEH); 1♀: Otjivarongo (cSEH); Gobabis-Aranos (cSEH); 1♂: Maltahohe (cSEH).

**Republic of South Africa.** 1♂, 3♀: Northern Cape Province, SW Kimberley, 13 km SW Ritchie (cHAC).

##### Distribution.

Southern Kenya, Namibia, Tanzania, Republic of South Africa.

### Comments on classification

Our study shows the following:

1. Termination (apex) of the aedeagus. We have found no difference in shape of the apex among species or subspecies within the same group, the shape is distinct only among species belonging to different groups. The apex of *Calosoma imbricatum* (narrower and sharper, Plate 3: Figs 1–2. Plate 4: Fig. 6) differs from those in all species of the *Calosoma senegalense* group (more blunt apex, Plate 3: Fig. 3. Plate 4: Figs 1–5). The apex in Jeannel’s holotype of *Calosoma aethiops* corresponds to that in the *Calosoma imbricatum* group (Plate 3), which does not support the opinion of Bruschi (2013) that *Calosoma aethiops* is a synonym of *Calosoma scabrosum roeschkei*.

2. Shape of inflated endophalus. We have not found an apparent difference either among species or subspecies belonging to the same group, or among species belonging to different groups (Plate 4: Figs 1–5). This result does not support the opinion that shape of the endophalus can be used to indicate assignments to to species or species groups.

3. Morphometric parameters.

A. Total length including mandibles (TL). In three measured holotypes (or lectotypes) are the values within the minimum and maximum intervals found in corresponding populations and sexes, whereas in the male holotype of *Calosoma aethiops* the value is outside of the interval. The TL value in the *Calosoma aethiops* holotype is closest to the values found in males of *Calosoma imbricatum hottentotum*, and lies within the interval found in females of that subspecies (all types are males, see Table 1a, Table 2). This fact supports our opinion that the holotype of *Calosoma aethiops* is an extremely large male of *Calosoma imbricatum hottentotum*.

**Table 1a.** Total length, intervals.

Taxon Holotype (HT), lectotype (LT)	**Total length** including mandibles in millimeters (TL). Interval of minimum and maximum value of TL measured in 10 specimens of the same sex is in parentheses. Yes (Y) – TL value of type is within interval. No (N) – TL value of type is outside of interval.	
*Calosoma scabrosum scabrosum* Chaudoir, 1843 (HT ♂)	25.5 (23.1–26.2 ♂♂)	Y
*Calosoma scabrosum roeschkei* Breuning, 1927 (HT ♂)	25.0 (22.0–28.5 ♂♂)	Y
*Calosoma imbricatum hottentotum* Chaudoir, 1852 (♂ LT Deuve 1978)	21.0 (17.5–21.3 ♂♂)	Y
*Calosoma aethiops* (Jeannel, 1940) (HT ♂)	21.5 (*Calosoma imbricatum hottentotum* ♂♂ 17.5–21.3 ♀♀ 19.2–23.0)	N

B. Maximum pronotal width to maximum head width including eyes ratio (WP/WH). The WP/WH in two measured holotypes (or lectotypes) is within the minimum and maximum intervals found in the pertinent populations and sexes. In the third taxon the WP/WH value of the holotype is outside of the interval in both sexes. In the holotype of *Calosoma aethiops* is the WP/WH value within the interval found in the corresponding sex (males) of *Calosoma scabrosum roeschkei* and also within the interval found in *Calosoma imbricatum hottentotum* females. Overall the WP/WH values found in the measured taxa is very variable in both species and sexes (Table 1b, Table 2), and in our opinion thus cannot be used as a criterion in species-level taxonomy.

**Table 1b.** Pronotal width to head width ratio, intervals.

Taxon Holotype (HT), lectotype (LT)	**Maximum pronotal width to maximum head width** inc. eyes ratio (WP/WH). Interval of minimum and maximum value of WP/WH measured in 10 specimens of the same sex is in parentheses. (Y/Y) – value of type is within interval. (N/N) – value of type is outside of interval. (N/Y) – value of type is outside of interval in males but within interval in females.	
*Calosoma scabrosum scabrosum* Chaudoir, 1843 (HT ♂)	1.50 (1.34–1.44 ♂♂) (1.35–1.48 ♀♀)	N/N
*Calosoma scabrosum roeschkei* Breuning, 1927 (HT ♂)	1.50 (1.29–1.63 ♂♂) (1.41–1.65 ♀♀)	Y/Y
*Calosoma imbricatum hottentotum* Chaudoir, 1852 (♂ LT Deuve 1978)	1.45 (1.33–1.50 ♂♂) (1.23–1.54 ♀♀)	Y/Y
*Calosoma aethiops* (Jeannel, 1940) (HT ♂)	1.32 (*Calosoma imbricatum hottentotum*) (*Calosoma scabrosum scabrosum*) (*Calosoma scabrosum roeschkei*)	N/Y N/N Y/N

Calosoma
Maximum pronotal width to its maximum length ratio (WP/LP). The WP/LP value in two measured holotypes (or lectotypes) is with exception of males of *Calosoma scabrosum scabrosum* within the minimum and maximum intervals found in the pertinent populations and sexes. In the holotype of *Calosoma aethiops* is the WP/WH value within the interval found in all compared species of both sexes. Overall the WP/LP values found in the measured taxa are quite non-specific in both species and sexes (Table 1c, Table 2), and in our opinion thus cannot be used as a criterion in species-level taxonomy.

**Table 1c.** Pronotal width to length ratio, intervals.

Taxon Holotype (HT), lectotype (LT)	**Maximum pronotal width to maximum pronotal length ratio** (WP/LP). Interval of minimum and maximum value of WP/LP measured in 10 specimens of the same sex is in parentheses. (Y/Y) – value of type is within interval. (N/N) – value of type is outside of interval. (N/Y) – value of type is outside of interval in males but within interval in females.	
*Calosoma scabrosum scabrosum* Chaudoir, 1843 (HT ♂)	1.65 (1.33–1.56 ♂♂) (1.52–1.67 ♀♀)	N/Y
*Calosoma scabrosum roeschkei* Breuning, 1927 (HT ♂)	1.67 (1.38–1.77 ♂♂) (1.47–1.74 ♀♀)	Y/Y
*Calosoma imbricatum hottentotum* Chaudoir, 1852 (♂ LT Deuve 1978)	1.74 (1.43–1.74 ♂♂) (1.55–1.84 ♀♀)	Y/Y
*Calosoma aethiops* (Jeannel, 1940) (HT ♂)	1.55 (*Calosoma imbricatum hottentotum*) (*Calosoma scabrosum scabrosum*) (*Calosoma scabrosum roeschkei*)	Y/Y Y/Y Y/Y

D. Maximum elytral length to its maximum width ratio (LE/WE). The WP/LP value in two measured holotypes (or lectotypes) is in both subspecies of *Calosoma scabrosum* within the minimum and maximum intervals found in the pertinent populations and sexes. In both sexes of *Calosoma imbricatum hottentotum* the value is outside the interval. In the holotype of *Calosoma aethiops* the value is within the interval found in both subspecies of *Calosoma scabrosum*. Overall the WP/LP values found in the measured taxa can be regarded as variable, namely in *Calosoma imbricatum hottentotum*. In our opinion they cannot be used as a criterion in species-level taxonomy (Table 1d, Table 2).

**Table 1d.** Elytral length to width ratio, intervals.

Taxon Holotype (HT), lectotype (LT)	**Maximum elytral length to maximum elytral width ratio** (LE/WE). Interval of minimum and maximum value of LE/WE measured in 10 specimens of the same sex is in parentheses. (Y/Y) – value of type is within interval. (N/N) – value of type is outside of interval. (N/Y) – value of type is outside of interval in males but within interval in females.	
*Calosoma scabrosum scabrosum* Chaudoir, 1843 (HT ♂)	1.44 (1.44–1.56 ♂♂) (1.38–1.53 ♀♀)	Y/Y
*Calosoma scabrosum roeschkei* Breuning, 1927 (HT ♂)	1.52 (1.39–1.57 ♂♂) (1.33–1.59 ♀♀)	Y/Y
*Calosoma imbricatum hottentotum* Chaudoir, 1852 (♂ LT Deuve 1978)	1.54 (1.33–1.50 ♂♂) (1.38–1.57 ♀♀)	N/Y
*Calosoma aethiops* (Jeannel, 1940) (HT ♂)	1.39 (*Calosoma imbricatum hottentotum*) (*Calosoma scabrosum scabrosum*) (*Calosoma scabrosum roeschkei*)	Y/Y N/Y Y/Y

**Table 2.** Intervals of all measurements in each taxon (intervals), in TL and LE/WE for both sexes separately and total.

*Calosoma scabrosum scabrosum*	23.1–26.2	23.1–26.2	23.4–25.7	1.35–1.63	1.33–1.67	1.38–1.56	1.44–1.56	1.38–1.53
*Calosoma scabrosum roeschkei*	22.0–28.5	22.0–28.5	24.1–27.9	1.29–1.65	1.38–1.77	1.33–1.59	1.39–1.57	1.33–1.59
*Calosoma senegalense*	23.0–30.0	23.0–29.3	24.9–30.0	1.22–1.62	1.30–1.73	1.45–1.56	1.45–1.55	1.45–1.56
*Ctenosta strandi*	25.2–30.3	25.2–29.0	26.4–30.3	1.32–1.44	1.46–1.62	1.44–1.64	1.50–1.64	1.44–1.64
*Calosoma imbricatum imbricatum*	17.0–22.0	17.0–20.4	18.2–22.0	1.33–1.43	1.41–1.68	1.26–1.57	1.39–1.53	1.26–1.57
*Calosoma imbricatum hottentotum*	17.5–23.0	17.5–21.3	19.2–23.0	1.33–1.54	1.43–1.84	1.33–1.57	1.33–1.50	1.38–1.57

**Map 1.**
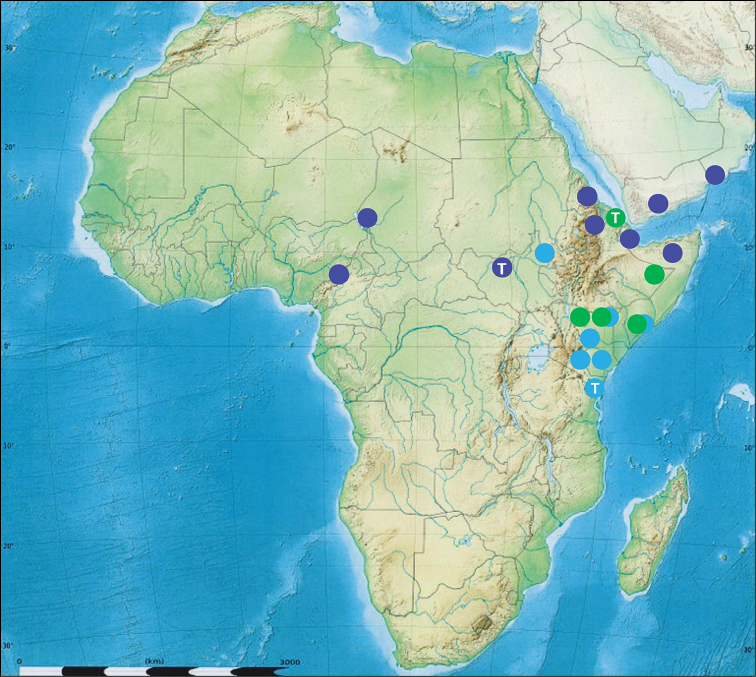
Geographic distribution of the *Calosoma scabrosum* and *Calosoma strandi* species subgroups (sensu Häckel 2012). T – the type locality. Dark blue discs – *Calosoma scabrosum* s. str. Chaudoir, 1843. Light blue discs – *Calosoma scabrosum roeschkei* Breuning, 1927. Green discs – *Calosoma strandi* Breuning, 1934.

The above data lead us to conclude that there are no convincing morphological differences between *Calosoma scabrosum scabrosum* and *Calosoma scabrosum roeschkei*. The only exception may possibly be the somewhat higher WP/LP ratio (Table 2) and lighter coppery coloration in most specimens of *Calosoma scabrosum roeschkei*. Since specimens of both taxa have never been found together, we assume that they belong to allopatric populations of one species that have yet to reach the state of full speciation. Therefore, we lower the status of *Calosoma roeschkei* (sensu Bruchi 2013) to a subspecies of *Calosoma scabrosum*. *Calosoma scabrosum roeschkei* occupies mainly the southern part of distribution of the species. The present data show the north – south distribution of both subspecies to have a virtually disjunct character (Map 1). But it is in our opinion important to realize that in no area have the described subspecies been found to occur together. It is therefore likely that the northern Sudan – Ethiopia borderland will continue to produce *Calosoma scabrosum roeschkei*. The describer (Breuning 1927: 186) of *Calosoma scabrosum roeschkei* wrote: [“This form (*roeschkei*) is due to its more robust head, less bulging eyes, wider, toward base more right-angled pronotum with more rounded hind angles, shallower basal pits, somewhat flatter, at shoulders broader and terminally more abruptly slanted elytra and dark to brownish bronze dorsal coloration with light brown, coppery-rimmed margins and foveae in primary intervals so conspicuous that I originally intended to describe it as a separate species. However, some individuals of this form (*roeschkei*) are clearly transitional to the typical form (*scabrosum*), with coloration remaining as the only constant character. Differences (of separate populations) from the nominotypical form show step-like transitions, for which reason I presently regard *Calosoma roeschkei* as a subspecies of *Calosoma scabrosum*” (original in German)].

Jeannel (1940: 128) did not see Breuning’s subspecies but treated it as a variety and remarked: [“Breuning’s variety *roeschkei* rather appears to be another (separate) species closely related to *Calosoma scabrosum*]”; he did not further comment on the taxon and placed specimens corresponding to Breuning’s description among African populations of “*Ctenosta orientale*” (Jeannel 1940: 128). In this connection, Jeannel and subsequent authors such as Rougemont (1976: 247) and Vigna Taglianti and Bruschi (1986: 21) solved taxonomic uncertainties by incorrect determination of specimens from eastern Africa as *Calosoma squamigerum* Chaudoir, 1869 (Häckel 2012: 57.). The holotype of *Calosoma squamigerum* is from Bengal (today either Bangladesh or West Bengal in India) and other specimens of the type series are from Coimbatore in the vicinity of Madras (today Tamilnadu State in southern India). The name *Calosoma squamigerum* was therefore synonymized with *Ctenosta orientale* (Jeannel 1940: 128).

Jeannel nevertheless realized that African populations identified as *Ctenosta orientale* (=*squamigerum*) most likely belong to another species, coined for them a new taxon, Ctenosta (s. str.) aethiops Jeannel, 1940, and included in the distribution of this taxon also populations corresponding to Breuning’s *Calosoma scabrosum roeschkei* (“Diré Daoua”, Jeannel 1940: 128). The only exception was a population from Eritrea (“Tessenei” 1940: 129), which he continued regarding as *Ctenosta orientale*. In the description of *Calosoma aethiops* Jeannel (1940: 127–128) wrote: [“if we regard *Calosoma aethiops* with its gular and labial setae as belonging to the genus *Caminara*, we can see that in reality it is a transitional species combining characters of *Caminara* and *Ctenosta*, an important species attesting to its assignment to the genus *Ctenosta*, which differs by reduction of the said setae and the type of sculpture placing it near the *Castrida*-*Caminara* lineage. The male mesotibiae have the setal brush prolonged as in *Caminara* and similar to that present in *Ctenosta*. The ventral side of the fourth male protarsomere is smooth]”.

Evident from Jeannel’s text are the difficulties he had in placing the new species in his system and in defining the “genera”. More recently some authors (Culot 1990, Bousquet et al. 2003, Bruschi 2013) regarded the genera as subgenera, but in our opinion Jeannel’s criteria do not allow to distinguish them. For instance Bruschi in the key does not adhere to Jeannel’s criteria (setae, elytral microsculpture) and originally separates *Caminara* from *Ctenosta* on the hind pronotal angles. In the key he (2013: 29) states: “13(14) always perceptible hind angles of pronotum – *Caminara*/*Campalita*; 14(13) very small and pointed hind angles of pronotum, in some cases quite obliterated”. In our opinion Jeannel’s genera are not valid, which is supported also by their discord with results of DNA analyses. With only one exception, all the subgenera were synonymized with the genus *Calosoma* s. str. (Häckel 2012: 56, 2013: 12). Jeannel’s difficulties in separating the “genera” *Ctenosta* and *Caminara* (according to us two close species groups of the genus *Calosoma*) reflect the confused composition of the type series of his *Calosoma aethiops*. Without examination of Jeannel’s holotype of *Calosoma aethiops*, most subsequent authors regarded all African populations similar to *Calosoma scabrosum*, with golden bronze coloration, as Calosoma (Ctenosta) aethiops (Vigna Taglianti and Bruschi 1986: 21, Culot 1990: 9, Lorenz 2005: 68).

Only Bruschi, first on the internet and later in print (2013: Plate 17: Figs 8, 9), published photos of the holotypes of *Calosoma scabrosum roeschkei* (Tanzania: Usambara) and *Calosoma aethiops* (Niger: Azbin). The holotype of *Calosoma scabrosum roeschkei* (Usambara, see Map 1) clearly is a species belonging to the *Calosoma senegalense* group, and we concur with Bruschi that in compliance with the priority principle the taxon must be ascribed to Breuning (the name *Calosoma scabrosum roeschkei* has priority over *Calosoma aethiops*, if the two are the same species).

However, in our opinion the specimen from Azbin (Jeannel’s holotype of *Calosoma aethiops*) looks different and does not belong to the *Calosoma senegalense* group (=Jeannel’s genus *Ctenosta*). Our comparisons of types and their aedeagi show that the holotype of *Calosoma aethiops* corresponds in shape, size and sculpture of the elytra, shape of the legs, and termination of the aedeagus to *Calosoma imbricatum hottentotum* Chaudoir, 1852. It belongs to another group (*Calosoma maderae* group, *Calosoma imbricatum* subgroup sensu Häckel, 2013: =Jeannel’s genus *Caminara*), which partially overlaps the distribution of the *Calosoma senegalense* group. Our conclusions are based chiefly on the different aedeagal morphology unequivocally shown by the photos (Plate 3, Figs 1, 2 versus Fig. 3). Futher documentation of morphological characters is not needed.

**Map 2.**
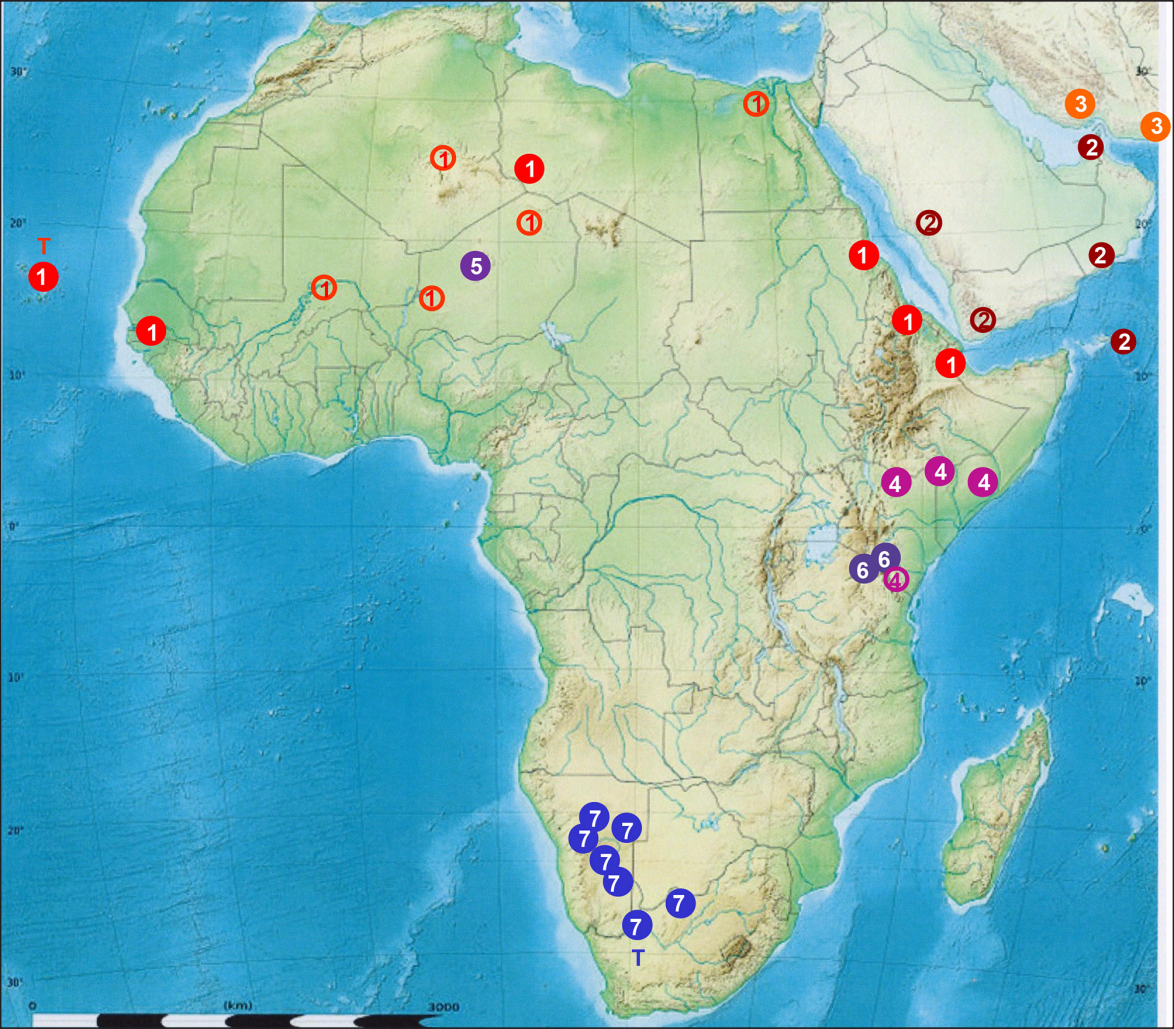
Geographic distribution of the *Calosoma imbricatum* species subgroup (sensu Häckel 2013) compared to Mandl‘s data (1970) and *Calosoma imbricatum hottentotum* Chaudoir, 1852. T – the type locality, ● (full disc) – recent records, ⚬ (empty disc) – Mandl‘s data **1–4** *Calosoma imbricatum* s. str. Klug, 1832 **5–7** *Calosoma imbricatum hottentotum* Chaudoir, 1852 **1** *Calosoma imbricatum* s. str. (sensu Mandl 1970) **2** *Calosoma imbricatum arabicum* (Motschulsky, 1865) sensu Mandl 1970 **3** *Calosoma imbricatum löffleri* Mandl, 1953 sensu Mandl 1970 **4** *Calosoma imbricatum linnavourii* Mandl, 1970 sensu Mandl 1970 **5** *Calosoma imbricatum hottentotum* (holotype of *Ctenosta aethiops* Jeannel, 1940) **6** *Calosoma imbricatum hottentotum* (*Calosoma imbricatum linnavourii* sensu Mandl 1970) **7** *Calosoma imbricatum hottentotum*. sensu Mandl 1970.

*Calosoma imbricatum* sensu lato is by a number of authors understood as a species with an extremely wide distribution reaching from Canary and Cape Verde Islands through the African Sahel belt, subsaharan Africa, Arabia, Iran and Pakistan to India and Bagladesh (Breuning 1927: 221–223, Jeannel 1940: 104–106, Mandl 1970: 61–63, see Map 2). The cited authors identify southern African populations of *Calosoma imbricatum* as the subspecies *Calosoma imbricatum hottentotum*, whose holotype comes from the Cape province (Chaudoir 1852: 99). *Calosoma imbricatum hottentotum* is usually regarded as cofined to southern Africa, and Mandl (1970: 61, 63) even named another subspecies, *Calosoma imbricatum linnavourii*, for populations from eastern Africa (northern Kenya, Somalia), which is a transitional form differing from the northern (Sahel-Arabian) nominotypical form (*Calosoma imbricatum imbricatum*) by wider pronotum. Populations from southern Kenya and northern Tanzania with more lighter coppery coloration approaching rather the southern African populations (*Calosoma imbricatum hottentotum*). Jeannel (1940: 105) is the only author who regarded also the Kenya population as *Calosoma imbricatum hottentotum*. Mandl (1970: 63) commented these occurrences as “Wahrnscheinlich gehören die folgende Orte zu dieser Subspecies [*Calosoma imbricatum linnavourii*]: Kenya Sultan Hamid zwischen Voi und Nairobi, die Jeannel für *hottentottum* angibt”, and Bruschi (2013) countered: „It seems that, contrary to the opinions of Mandl (1970: 61) that attributed this citation to his *C imbricatumlinnavourii*, Jeannel is right since in south western Kenya *C imbricatumhottentotum* is really present“. In our opinion it is evident that the northern limit of *Calosoma imbricatum hottentotum* is vague and hosts a number of forms transitional to the nominotypical *Calosoma imbricatum imbricatum*. It is therefore possible that Jeannel’s type of *Calosoma aethiops* from Azbin (today northern Niger) belongs to one of the transitional populations and the locality is in fact correct (see Map 2). In our opinion Jeannel‘s type series of *Calosoma aethiops* contains the holotype that we regard as *Calosoma imbricatum* (most likely the subspecies *hottentotum*) and specimens from other populations belonging to some of the subspecies of *Calosoma scabrosum*, mostly to *Calosoma scabrosum roeschkei*.

Comparison of the types of *Calosoma scabrosum scabrosum* and *Calosoma scabrosum roeschkei* in our opinion also confirms the original Breuning’s idea of one species with two terminal forms and a number of transitional forms between them (Plate 1: Figs 1–2). Another taxonomical inaccuracy was caused by Häckel et al. (2005: 2), who believed that populations newly discovered on the Arabian peninsula (Oman, Yemen, see Map 1) represent a different species (*Calosoma jakli*, Plate 2. Fig. 2). Eventually, after comparison with the type of *Calosoma scabrosum scabrosum* it became clear that the Arabian specimens agree with the type (Häckel et al. 2010: 11), and today they are included in the distribution of the nominotypical subspecies *Calosoma scabrosum* s. str. (Müller 1977, Häckel 2012: 57).

Below we present morphometric tables comparing populations of *Calosoma scabrosum roeschkei* (hitherto labeled as *Calosoma aethiops*) with specimens of *Calosoma scabrosum scabrosum* the Horn of Africa (Djibouti) and the Arabian peninsula (Oman, Yemen). The tables also compare the noted populations of *Calosoma scabrosum* with specimens of *Calosoma imbricatum hottentotum* from southern and eastern Africa and *Calosoma imbricatum imbricatum* from Afrotropical Region. In this connection we consider it important that no known locality has produced sympatrically living *Calosoma scabrosum scabrosum* and *Calosoma scabrosum roeschkei* (see Map 1). However, at at least one locality (Kenya: North-Eastern Province, El Wak, see Map 1) has produced *Calosoma scabrosum* (ssp. *roeschkei*) together with *Calosoma imbricatum* (ssp. *hottentotum*) and another species of the *Calosoma senegalense* group (*Ctenosta strandi* Breuning). Our conclusion therefore is that Breuning’s (1928: 185) original idea is valid, and consequently we demote *Calosoma roeschkei* sensu Bruschi (2013: 129) to a subspecies of *Calosoma scabrosum*.

The subgeneric placement of *Calosoma imbricatum*, *Calosoma* in the subgenus *Calosoma* follows the recently proposed classification supported by results of DNA analyses (Su et al. 2005, Häckel 2012, 2013).

## Supplementary Material

XML Treatment for Calosoma (Calosoma) planicolle

XML Treatment for Calosoma (Calosoma) scabrosumscabrosum

XML Treatment for Calosoma (Calosoma) scabrosumroeschkei

XML Treatment for Calosoma (Calosoma) senegalense

XML Treatment for Calosoma (Calosoma) strandi

XML Treatment for Calosoma (Calosoma) imbricatumimbricatum

XML Treatment for Calosoma (Calosoma) imbricatumhottentotum
